# Sox9 confers stemness properties in hepatocellular carcinoma through Frizzled-7 mediated Wnt/β-catenin signaling

**DOI:** 10.18632/oncotarget.8835

**Published:** 2016-04-19

**Authors:** Carmen Oi-Ning Leung, Wing-Nga Mak, Alan Ka-Lun Kai, Kwan-Shuen Chan, Terence Kin-Wah Lee, Irene Oi-Lin Ng, Regina Cheuk-Lam Lo

**Affiliations:** ^1^ Department of Pathology, Li Ka Shing Faculty of Medicine, The University of Hong Kong, Pok Fu Lam, Hong Kong; ^2^ State Key Laboratory for Liver Research, The University of Hong Kong, Pok Fu Lam, Hong Kong

**Keywords:** Sox9, liver cancer, tumor-initiating cells

## Abstract

Sox9, an SRY-related HMG box transcription factor, is a progenitor/precursor cell marker of the liver expressed during embryogenesis and following liver injury. In this study, we investigated the role of Sox9 and its molecular mechanism with reference to stemness properties in hepatocellular carcinoma (HCC). Here, we observed upregulation of Sox9 in human HCC tissues compared with the non-tumorous liver counterparts (*p* < 0.001). Upregulation of Sox9 transcript level was associated with poorer tumor cell differentiation (*p* = 0.003), venous invasion (*p* = 0.026), advanced tumor stage (*p* = 0.044) and shorter overall survival (*p* = 0.042). Transcript levels of Sox9 and CD24 were positively correlated. Silencing of Sox9 in HCC cells inhibited *in vitro* cell proliferation and tumorsphere formation, sensitized HCC cells to chemotherapeutic agents, and suppressed *in vivo* tumorigenicity. In addition, knockdown of Sox9 suppressed HCC cell migration, invasion, and *in vivo* lung metastasis. Further studies showed that Sox9 endowed stemness features through activation of Wnt/β-catenin signaling, which was confirmed by the partial rescue effect on tumorigenicity and self-renewal upon transfection of active β-catenin in Sox9 knockdown cells. By ChIP and luciferase promoter assays, Frizzled-7 was identified to be the direct transcriptional target of Sox9. In conclusion, Sox9 confers stemness properties of HCC through Frizzled-7 mediated Wnt/β-catenin pathway.

## INTRODUCTION

Sox9 is a member of the sex-determining region Y (SRY)-related high-mobility-group box transcription factors. Sox proteins are heavily involved in human developmental process and regulate lineage restriction, cell differentiation and stem cell properties [1]. Among the Sox family, Sox9 promotes testis differentiation and cartilage formation. In the liver, Sox9 is expressed in the progenitor/precursor cells during embryogenesis and during hepatocyte regeneration following liver injury [2, 3].

Sox9 overexpression was found in human cancers including prostate, lung and colon cancers, and is associated with more aggressive clinicopathological features and poorer prognosis [4–6]. Silencing of Sox9 inhibits cell growth and clonogenicity in lung cancer [7]. Abrogation of Sox9 blocks initiation of prostate cancer [8]. In esophageal cancer, Sox9 promotes tumorsphere formation and invasive capacity; and in colon cancer it enhances tumorigenicity [9, 10]. On signaling pathways, Sox9 binds directly to BMI1 in colon cancer [10], co-operates with slug to induce mammary stem cells in the breast [11], and drives tumorigenesis through the ERBB pathway in the pancreas [12].

In hepatocellular carcinoma (HCC), a recent study showed that Sox9 overexpression is associated with higher tumor stage and tumor grade and poorer survival [13]. While being a liver progenitor cell marker, the potential role and molecular mechanisms of Sox9 in conferring stem cell-like properties in HCC remain to be further elucidated. Cancer stem cells (CSCs)/tumor-initiating cells (T-ICs) in solid cancers share many of the characteristics of normal stem cells and drive tumor initiation, self-renewal, chemoresistance, tumor recurrence and metastasis [14, 15]. In the liver, the existence and importance of CSC/T-IC have also been substantiated [16–18]. In this study, we aimed at investigating the functional significance of Sox9 and its downstream signaling pathway with specific reference to stemness features in HCC.

## RESULTS

### Sox9 is overexpressed in human HCC

**Figure 1:**
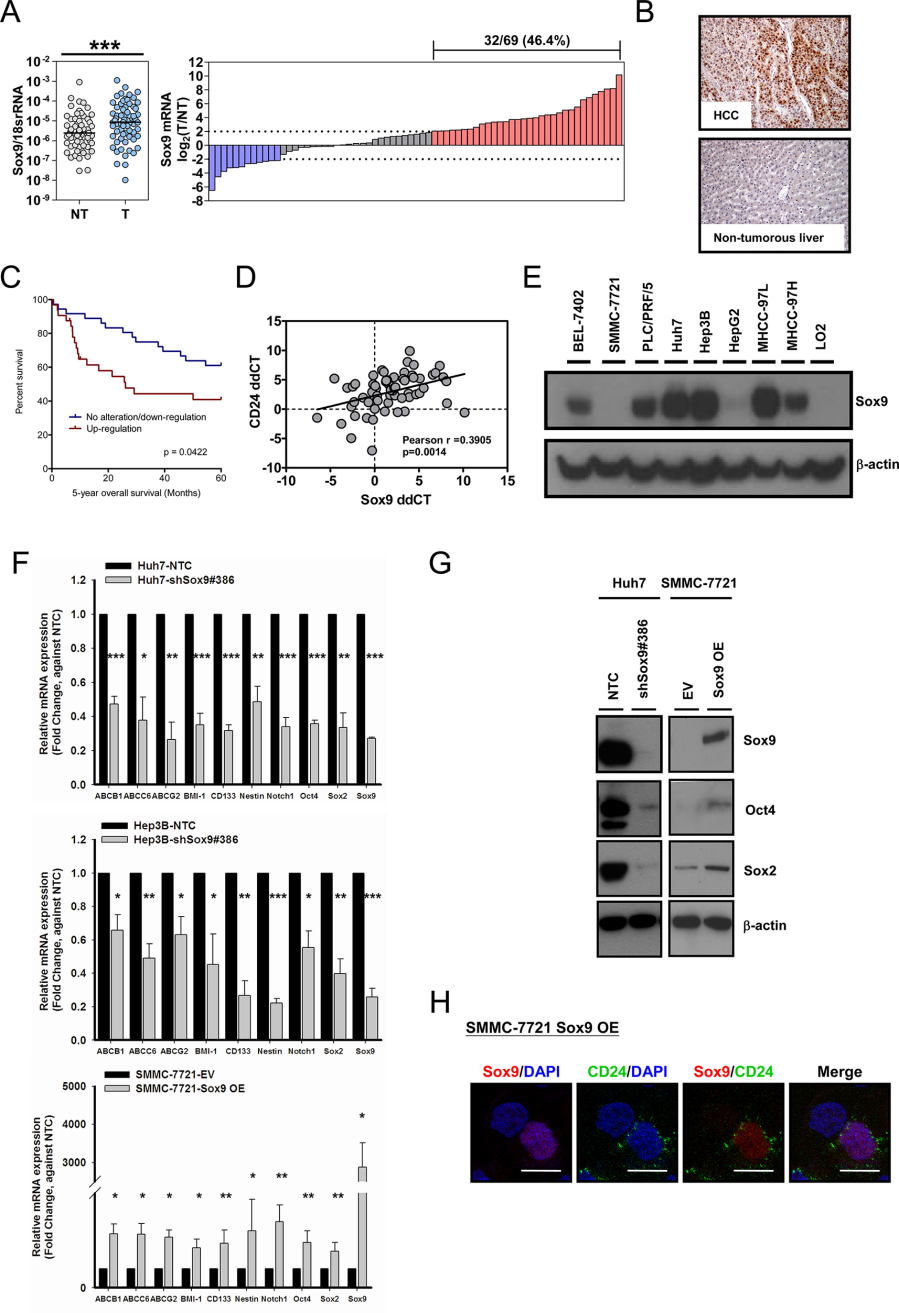
Sox9 is upregulated in human HCC and Sox9 expression is associated with expression of stemness markers *in vitro* (**A**) Sox9 was significantly upregulated in HCC tumor (T) versus non-tumorous liver (NT) tissues by qPCR (*n* = 69; ***p < 0.001, Wilcoxon signed-rank test). Upregulation of Sox9 was found in (32/69) 46.4% of primary HCC cases. Data were presented as the log_2_ ratio of Sox9 mRNA level in HCC tissues, as compared to the corresponding non-tumorous liver tissues. Upregulation was defined as log_2_ (T/NT) ≥ 2. (**B**) Representative images of immunohistochemical staining for Sox9 in clinical HCC samples demonstrated the overexpression at protein level in HCC tissues, while the non-tumorous liver counterparts showed no staining among the hepatocytes. (**C**) Kaplan-Meier survival curve of overall survival in HCC patients with reference to Sox9 expression. Patients with Sox9 upregulation had lower overall 5-year overall survival rate (*p* = 0.0422, Kaplan-Meir Log-rank test). (**D**) Expressions of CD24 and Sox9 were positively correlated in clinical HCC samples. Expression level of CD24 and Sox9 in HCC samples was represented by ddCT (Tumor _18srRNA CT - Gene of interest CT_^−^ Non-tumor _18srRNA CT_^−^_Gene of interest CT_) (*n* = 64, Pearson *r* = 0.3905, *p* < 0.01, Pearson's correlation). (**E**) Sox9 was expressed in multiple HCC cell lines by Western blotting, including BEL-7402, PLC/PRF/5, Huh7, Hep3B, MHCC-97L and MHCC-97H. It was not detectable in immortalized liver cell line LO2. (**F**) Knockdown of Sox9 (clone #386) in Huh7 and Hep3B diminished the expression of multiple chemoresistance-related and stemness-associated genes when compared with non-target control (NTC), while overexpression of (OE) Sox9 in SMMC-7721 reversed the expressions compared with empty vector group (EV) (*n* = 3, *p < 0.05, **p < 0.01 & ***p < 0.001, *t* test). The data were presented as mean ± SD. (**G**) Protein level of two stemness genes, Oct4 and Sox2, were suppressed in Huh7 upon silencing of Sox9 and enhanced upon forced-expression of Sox9 in SMMC-7721. (**H**) CD24 expression was enhanced upon transient Sox9 overexpression in SMMC-7721 while it was not expressed in untransfected cell (without Sox9 signal) by immunofluorescent staining (Scale bar: 50 μm).

By qPCR we assessed the transcript levels of Sox9 in the tumor tissues and the corresponding non-tumorous liver tissues in 69 human HCC samples. Sox9 was overexpressed in HCC tissues compared to the non-tumorous counterpart (*p* < 0.001). Sox9 upregulation (tumor/non-tumor ≥ 4) was observed in 32 cases (46.4%) (Figure 1A). Sox9 overexpression was also demonstrated at protein level by immunohistochemistry (IHC). Positive staining was detected in HCC cells while the hepatocytes in the non-tumorous tissue showed no staining (Figure 1B). A significant correlation between Sox9 mRNA and protein overexpression was observed (*p* = 0.0008) (Supplementary Table S1). The expression data in the clinical cohort were subjected to statistical correlation with various clinicopathological parameters in our database. Upregulation of Sox9 (by qPCR) in HCC was associated with poorer tumor cell differentiation (*p* = 0.003), venous invasion (*p* = 0.026), higher tumor stage (*p* = 0.044) and shorter overall survival (Table 1 and Figure 1C). Furthermore, Sox9 transcript level in HCC tissues is positively correlated with that of CD24, our previously characterized liver T-IC marker [17] (Figure 1D). We also examined the immunohistochemical expression of stemness markers CK19, AFP and EpCAM in the clinical cohort. Seventeen cases (of 67 examined) showed positive CK19 staining. The percentage of positivity is similar to that reported previously [19]. Interestingly, all CK19+ cases were Sox9+, and 14 of the 17 CK19+ cases were demonstrating high Sox9 immunoexpression. In addition, among the Sox9+ subset, majority of AFP+ and EpCAM+ cases (18/22 and 17/22 for AFP and EpCAM respectively) was associated with a high Sox9 immunoexpression (Supplementary Figure S1 and Supplementary Table S2). By Western blotting in a panel of HCC cell lines, Sox9 was abundantly expressed in BEL-7402, PLC/PRF/5, Huh7, Hep3B, MHCC-97L and MHCC-97H cell lines, while the immortalized normal liver cell line LO2 showed no Sox9 expression (Figure 1E).

**Table 1:** Clinicopathological correlation of Sox9 expression in human HCCs

Parameter	Sox9 T:NT < 4	Sox9 T:NT ≥ 4	*p* value (Fisher's Exact Test)
Gender			
Male	27	26	0.569
Female	10	6	
Tumor size			
≤ 5 cm	17	10	0.219
> 5 cm	19	22	
Venous invasion			
Absent	20	8	0.026*
Present	17	24	
Tumor encapsulation			
Absent	21	25	0.066
Present	15	6	
Microsatellite formation			
Absent	19	11	0.141
Present	16	21	
Direct liver invasion			
Absent	19	19	1.000
Present	11	11	
Cellular differentiation			
Edmondson (I–II)	22	8	0.003**
Edmondson (III–IV)	13	24	
pTNM stage			
I/II	17	7	0.044*
III/IV	19	24	
Non-tumorous liver			
Normal & chronic hepatitis	17	15	1.000
Cirrhosis	20	17	
Serum HBsAg			
Negative	6	5	1.000
Positive	29	27	

Upregulation of Sox9 in HCC was associated with poorer tumor cell differentiation (*p* = 0.003), presence of venous invasion (*p* = 0.026) and advanced tumor stage (*p* = 0.044).

### Sox9 expression in HCC is associated with expression of stemness markers *in vitro*

In order to examine the phenotypic features and functional roles of Sox9, we established stable Sox9 knockdown clones by a lentiviral-based approach in high Sox9-expressing Huh7 and Hep3B cells, and a transient Sox9 overexpression in low Sox9-expressing SMMC-7721. Upon knockdown of Sox9 in HCC cells (Huh7 and Hep3B), we observed a downregulation of stemness-associated genes including BMI-1, CD133, Sox2, Nestin, Notch1 and Oct4, and chemoresistance-related genes (ABCB1, ABCC6, ABCG2) by qPCR compared with non-target control (NTC). Overexpression of Sox9 in SMMC-7721 brought about the opposite effects (Figure 1F). The effect of Sox9 on Sox2 and Oct4 expression was further supported by Western blotting (Figure 1G). Besides, co-expression of CD24 in Sox9-expressing SMMC-7721 cells was observed by immunofluorescence (IF) staining (Figure 1H). These findings suggest that Sox9 is associated with stemness features in HCC.

### Silencing of Sox9 reduces T-IC features of HCC cells

**Figure 2:**
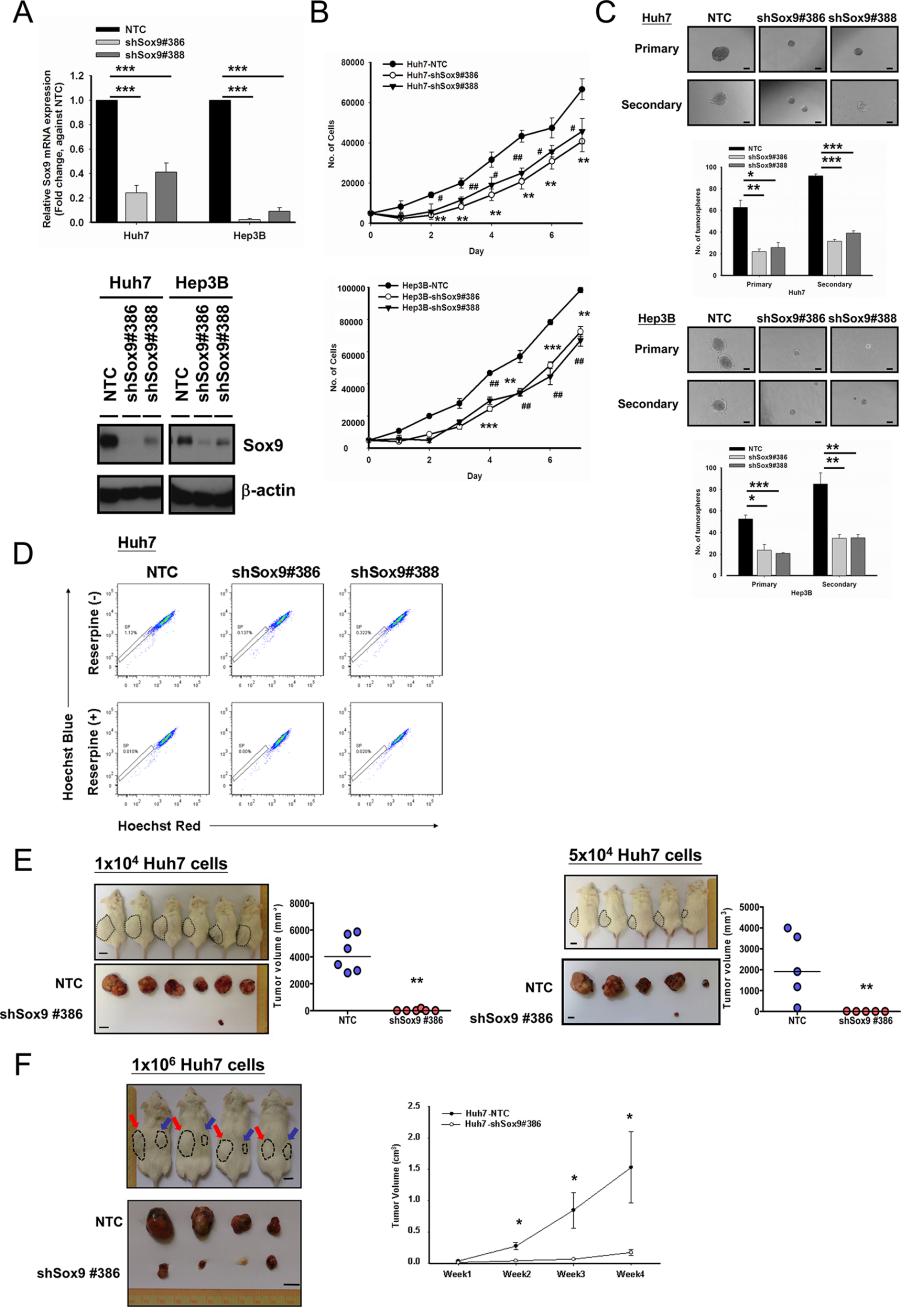
Silencing of Sox9 inhibits cell proliferation, tumorsphere formation and *in vivo* tumorigenicity in HCC (**A**) Two Sox9 stable-knockdown clones (#386 and #388) were established in Huh7 and Hep3B using lentiviral-based shRNA approach. Both Sox9 mRNA (*n* = 3, ***p < 0.001, *t* test) and protein levels were suppressed. (**B**) Knockdown of Sox9 inhibited cell proliferation in both Huh7 and Hep3B cells, when compared with NTC (*n* = 3, ^#^ *p* < 0.05, **/^##^ *p* < 0.01 & ***p < 0.001, *t* test). (**C**) Knockdown of Sox9 reduced the number and size of the primary and secondary tumorspheres comparing with non-target control (NTC) in both Huh7 and Hep3B cell lines at Day 10 and Day 15, respectively (*n* = 3, *p < 0.05, **p < 0.01 & ***p < 0.001, *t* test; Scale bar: 50 μm). The data were presented as mean ± SD. (**D**) Knockdown of Sox9 suppressed the side population (SP, shSox9#386: 0.137% & shSox9#388: 0.322%) when compared with non-target control (NTC) (1.12%) using Hoechst 33342 labeling. Reserpine sensitivity (lower panel) was used to verify the SPs and to determine optimal staining conditions. (**E**) Silencing of Sox9 suppressed tumorigenicity *in vivo* in limited dilution manner of 1 × 10^4^ and 5 × 10^4^ Huh7 cells. Knockdown of Sox9 decreased tumor volume compared with non-target control (NTC) group (Black dotted circle: NTC; **p < 0.01, Mann-Whitney's *U* test; Scale bar: 1 cm). Suppression of Sox9 showed a decrease in tumor incidence with both cell numbers injected. (**F**) Huh7 NTC and Huh7 shSox9 cells were injected subcutaneously into NOD/SCID mice (4 mice per group, 1 × 10^6^ cells injected per mouse). Mice were sacrificed at week 4. Knockdown of Sox9 significantly suppressed HCC tumor growth and volume comparing with non-target control (NTC) group (Red arrow: NTC; Blue arrow: shSox9#386; *p < 0.05; Scale bar: 1 cm). The data were presented as mean ± SD.

Since expression of Sox9 in HCC is associated with that of stemness-associated genes, we performed various T-IC assays through suppressing Sox9 expression by a lentiviral-based approach (Figure 2A). Silencing of Sox9 (Huh7 and Hep3B) decreased HCC cell proliferation (Figure 2B). With non-adherent sphere formation assay, the numbers of tumorspheres were significantly decreased in the Sox9-knockdown clones compared with NTC, and size of the tumorspheres was also reduced in both primary and secondary generations (Figure 2C). Silencing of Sox9 suppressed the side population in Huh7 cells (Figure 2D). In order to validate our *in vitro* findings in a more biological environment, we performed subcutaneous inoculation in NOD/SCID mice to study the functional effects of Sox9. Stable knockdown of Sox9 suppressed tumorigenicity *in vivo* in a limited dilution manner (Figure 2E). Prolonged tumor latency period was also observed (Supplementary Figure S2). Through injection of 1 × 10^6^ Huh7 cells, the tumor volume was significantly lower in shSox9 group at weeks 2–4 when compared with NTC group (Figure 2F).

### Sox9 confers chemoresistance in HCC

**Figure 3:**
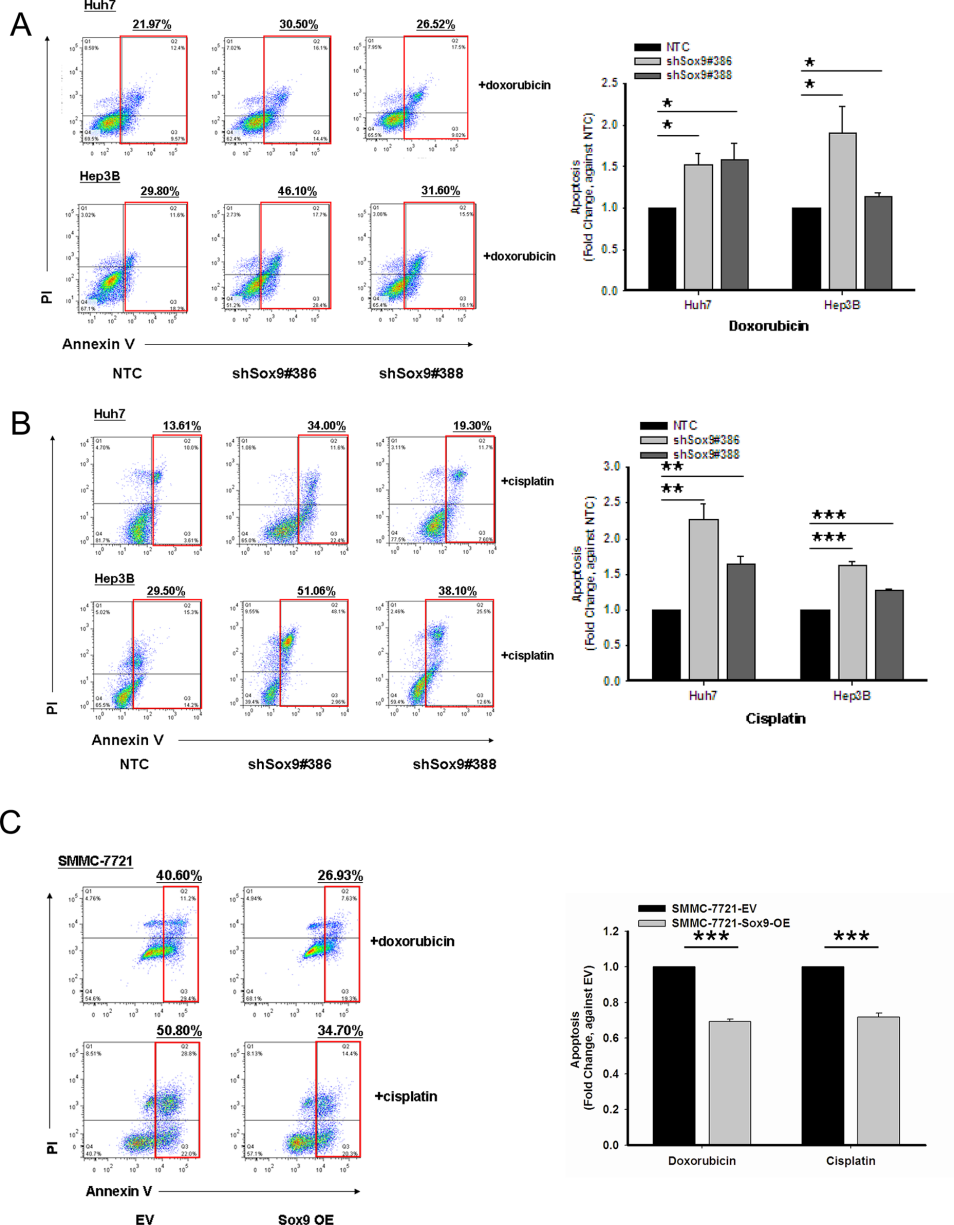
Sox9 confers chemoresistance in HCC Cells with stably knockdown of Sox9 were more chemosensitive to both (**A**) doxorubicin at 0.5 μg/mL for Huh7 and 1.5 μg/mL for Hep3B (*n* = 3, *p < 0.05, *t* test), and (**B**) cisplatin at 5 μg/mL for Huh7 and 10 μg/mL for Hep3B when compared with non-target control (NTC) group after 24-hour treatment at serum free condition (*n* = 3, **p < 0.01 & ***p < 0.001, *t* test). Representative Annexin V-PI flow analysis plots of each treatment were shown. (**C**) Sox9-overexpressing SMMC-7721 cells were more chemoresistant to both doxorubicin at 1 μg/mL and cisplatin 5 μg/mL when compared with empty vector (EV) group (*n* = 3, ***p < 0.001, *t* test). Representative Annexin V-PI flow analysis plots of each treatment were shown.

Our experiments showed that silencing of Sox9 inhibits tumorsphere formation *in vitro* and tumorigenicity *in vivo*. Chemoresistance is one of the major characteristics of T-ICs [16]. We found that knockdown of Sox9 downregulates the expression of multiple chemoresistance-related genes (Figure 1F). Hence we proceeded to study how Sox9 might alter chemosensitivity of HCC cells by apoptotic assays with Annexin V/PI staining. Silencing of Sox9 in HCC cells (Huh7 and Hep3B) increased the number of cells in apoptotic phase compared with NTC when treated with doxorubicin or cisplatin, and sensitized HCC cells to these chemotherapeutic agents. The opposite effects were observed upon forced expression of Sox9 (Figure 3). The results suggest that Sox9 confers chemoresistance in HCC cells.

### Knockdown of Sox9 inhibits cell migration, invasion *in vitro* and metastasis of HCC *in vivo*

**Figure 4:**
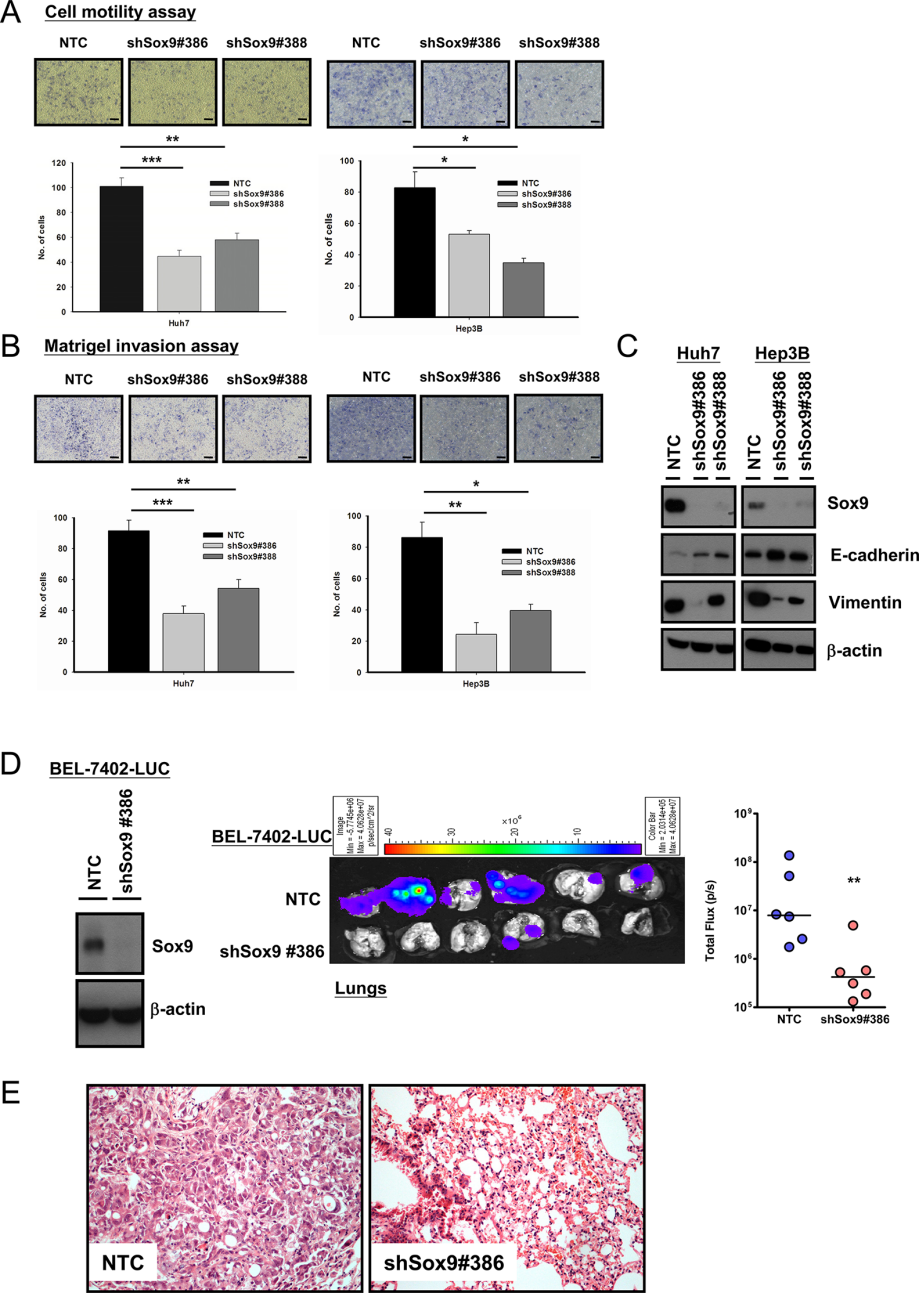
Silencing of Sox9 inhibits cell migration, invasion *in vitro* and metastasis of HCC *in vivo* Knockdown of Sox9 inhibited (**A**) cell migration ability and (**B**) invasiveness in both Huh7 and Hep3B cells, when compared with non-target control (NTC) (*n* = 3, *p < 0.05, **p < 0.01 & ***p < 0.001, *t* test; Scale bar: 50 μm). (**C**) Protein expression of E-cadherin was upregulated while vimentin was downregulated upon silencing of Sox9 as demonstrated by Western blotting, signifying inhibition of epithelial-mesenchymal transition with knockdown of Sox9. (**D**) Sox9 stable-knockdown clone and NTC were established in BEL-7402 luciferase-labeled cells using lentiviral-based shRNA approach. The efficiency of Sox9 silencing was confirmed by Western blotting. *Ex vivo* xenogen imaging of lungs at 12th week upon injection of either Sox9 silenced or NTC luciferase-labeled BEL-7402 through tail veins of NOD/SCID mice (6 mice per group, 1 × 10^6^ cells injected per mouse). Quantification of luciferase signals showed significant difference between the shSox9 and NTC groups (**p < 0.01, Mann-Whitney's *U* test). (**E**) Hematoxylin and eosin staining of mouse lung tissues from NTC and shSox9#386 groups indicated the suppression of pulmonary metastasis upon silencing of Sox9. Representative images were shown (Magnification: 200×).

By statistical correlation, Sox9 upregulation is associated with venous invasion and advanced tumor stage (Table 1). Hence we proceeded to confirm whether Sox9 facilitates HCC metastasis *in vitro* and *in vivo*. Silencing of Sox9 (Huh7 and Hep3B) decreased HCC cell migration (Figure 4A) and invasion (Figure 4B). With Western blotting and IF study, knockdown of Sox9 suppressed epithelial-mesenchymal transition (EMT) as demonstrated by upregulation of E-cadherin and downregulation of vimentin expressions (Figure 4C and Supplementary Figure S3). Next, we established the NTC and Sox9 knockdown clones using luciferase labeled-BEL-7402 with lentiviral-based approach. The efficiency was validated using Western blotting. We then employed an experimental metastasis model in NOD/SCID mice to investigate the role of Sox9 in HCC metastasis. At 12th week after tail vein injection, the lung metastasis rate in shSox9 group (BEL-7402-LUC) was lower (1/6) than that of NTC (6/6). Quantification of the corresponding luciferase signals further demonstrated a significant difference between the two groups (Figure 4D), and the lung tissues were analyzed by histological examination (Figure 4E).

### Sox9 endows stemness features in HCC through the canonical Wnt pathway

**Figure 5:**
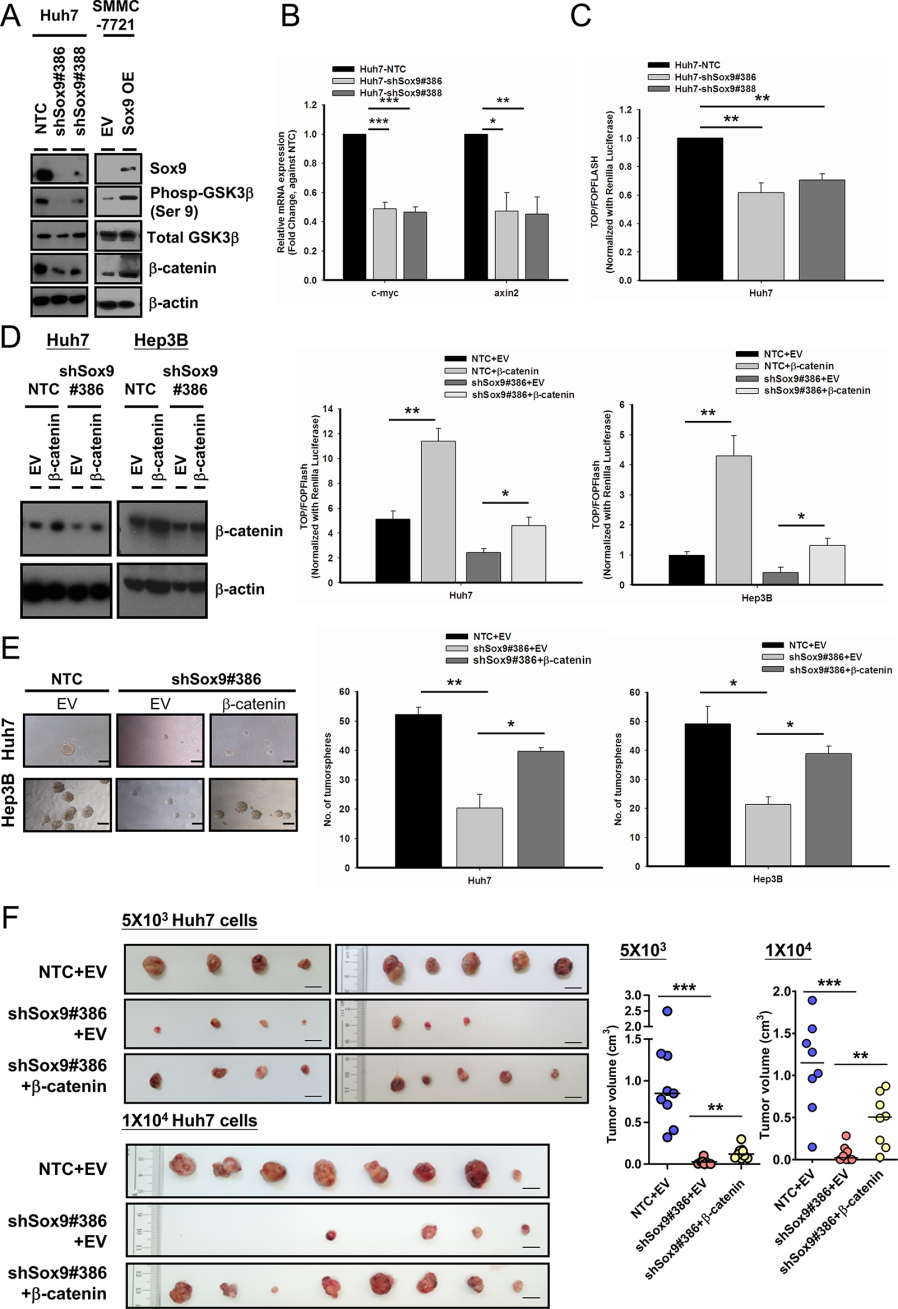
Sox9 enhances stemness features of HCC through Wnt/β-catenin signaling (**A**) Silencing of Sox9 downregulated the expression of phosphorylated (Ser9)-GSK3β and total β-catenin in Huh7 using Western blotting. The reversed effects were observed upon Sox9 overexpression in SMMC-7721. (**B**) Sox9 silencing (Huh7) suppressed the mRNA levels of c-myc and axin2. (*n* = 3, *p < 0.05, **p < 0.01 & ***p < 0.001, *t* test). (**C**) Knockdown of Sox9 suppressed the activation of Wnt/β-catenin signaling cascade in Huh7 using TOP/FOPFLASH luciferase assay (*n* = 3, **p < 0.01, *t* test). (**D**) Stable expression of β-catenin was enforced in shSox9 clone (#386) in Huh7 and Hep3B and validated using Western blotting. The suppression of Wnt/β-catenin signaling activity was partially rescued in shSox9 knockdown cells upon enforcement of β-catenin when compared with empty vector (EV) group (*n* = 3, *p < 0.05, **p < 0.01, *t* test). (**E**) The suppression of tumorsphere formation ability through knockdown of Sox9 was partially rescued upon β-catenin enforcement (*n* = 3, *p < 0.05, **p < 0.01, *t* test; Scale bar: 100 μm). The data were presented as mean ± SD. (**F**) β-catenin or EV-expressing-shSox9 silencing and EV-expressing Huh7-NTC were subcutaneously inoculated in NOD/SCID mice using 5 × 10^3^ and 1 × 10^4^ cells for 70 and 60 days, respectively. Upon β-catenin enforcement, the tumorigenicity of Sox9 silenced cells was partially rescued (*n* = 9, results from two independent experiments for 5 × 10^3^ group; *n* = 8 for 1 × 10^4^ group, **p < 0.01 & ***p < 0.001, Mann-Whitney's *U* test; Scale bar: 1 cm).

By *in vitro* and *in vivo* experiments, we showed that Sox9 confers stemness features and metastatic capability of HCC cells. Next, we wished to elucidate the downstream signaling pathway of Sox9 that gives rise to these features. The interaction between Sox9 and the canonical Wnt pathway in various human processes has been described. Physiologically, Sox9 degrades β-catenin in chondrogenesis [20] while in pancreatic development Sox9 represses β-catenin degradation [21]. In both breast cancer and glioma, Sox9 facilitates Wnt/β-catenin signaling [22, 23]. Thus, the effect of Sox9 on Wnt/β-catenin pathway may vary in different cellular contexts and biological processes. In this connection, we proceeded to determine whether Sox9 confers stem cell-like phenotypes in HCC through the Wnt/β-catenin pathway. We first examined the expression of key target molecules of the canonical Wnt pathway, pGSK3β and β-catenin, in HCC cells with altered Sox9 expression. We observed that knockdown of Sox9 suppressed the expression of phosphorylated (Ser9)-GSK3β and β-catenin, while total GSK3β level remained unchanged (Figure 5A). The expressions of axin2 and c-myc were also downregulated (Figure 5B and Supplementary Figure S4A). The reversed effects were observed with Sox9 transient overexpression (Figure 5A and Supplementary Figure S4B). In addition, silencing of Sox9 resulted in a decrease in Wnt/β-catenin pathway activation by TOP/FOPFlash reporter assay (Figure 5C and Supplementary Figure S4C). To further clarify the above findings, we performed rescue experiments by stably expressing constitutively active β-catenin plasmids (Plasmid 16520, Addgene, Cambridge, MA, USA) [24] in Sox9 knockdown cells. The effect with TOP/FOPFlash reporter assay in Sox9 silencing clones (Huh7 and Hep3B) was abrogated upon β-catenin forced expression (Figure 5D). Forced expression of β-catenin partially rescued the effect of Sox9 silencing on tumorsphere formation assays (Figure 5E), and similarly the effects on tumorigenicity, tumor volume and tumor latency period *in vivo* (Figure 5F and Supplementary Figure S4D). Hence we believe that the canonical Wnt pathway is mediating the downstream effect of Sox9 in HCC.

### Sox9 activates canonical Wnt pathway through direct binding with Frizzled-7

**Figure 6:**
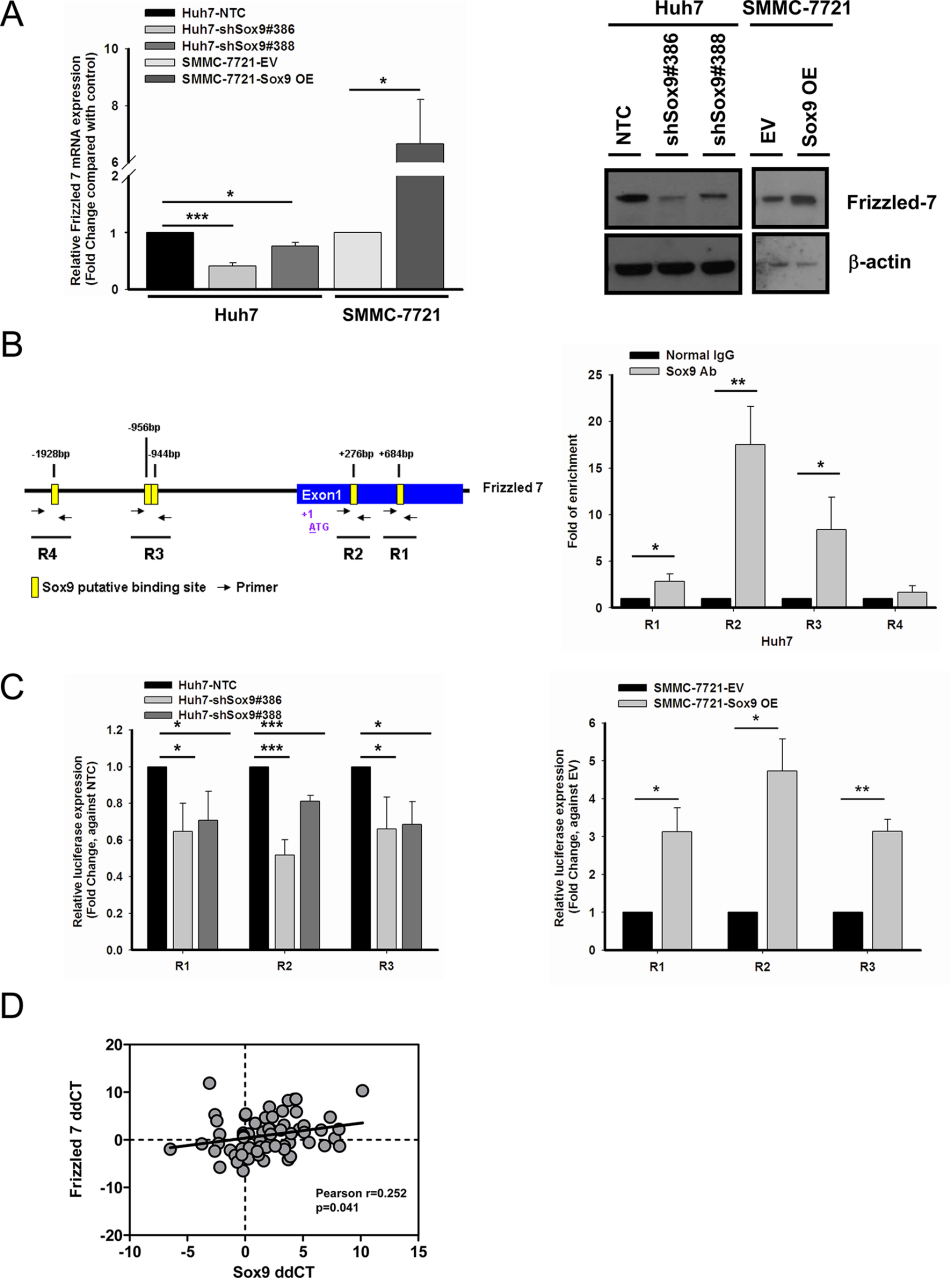
Frizzled-7 (FZD7) is a transcriptional target of Sox9 in HCC (**A**) FZD7 was suppressed at mRNA (*n* = 3, *p < 0.05 & ***p < 0.001, *t* test) and protein levels in Sox9 knockdown Huh7. The reversed effects were observed upon Sox9 overexpression in SMMC-7721. The data were presented as mean ± SD. (**B**) Chromatin immunoprecipitation (ChIP) assay using Sox9 antibody and normal rabbit IgG in Huh7. Schematic diagram indicated the putative binding sites of Sox9 (+684 bp as R1, +276 bp as R2, −944 bp & −956 bp as R3, −1928 bp as R4) on FZD7 promoter and exon 1 region (Yellow box: Sox9 putative binding site; Black arrow: primer for ChIP assay). Sox9 bound to R1, R2 and R3, but not R4 in Huh7 cells (*n* = 3, *p < 0.05 & **p < 0.01, *t* test). (**C**) A significant decrease of signals was detected in silenced Sox9 Huh7 comparing with non-target control (NTC) group, while the signals were upregulated in Sox9-overexpressing SMMC-7721 comparing with EV transfected group using luciferase reporter assay in R1, R2 and R3. (*n* = 3, *p < 0.05, **p < 0.01 & ***p < 0.001, *t* test). The data were presented as mean ± SD. (**D**) A positive correlation between FZD7 and Sox9 mRNA expressions was found in clinical HCC samples. Expression level of FZD7 and Sox9 in paired HCC samples was represented by ddCT (Tumor _18srRNA CT - Gene of interest CT_- Non-tumor _18srRNA CT - Gene of interest CT_) (*n* = 66, Pearson *r* = 0.252, *p* < 0.05, Pearson's correlation).

Having consolidated the above findings, we attempted to extend our investigation on identifying the transcriptional target of Sox9 in the canonical Wnt pathway. With reference to the ChIP-sequencing data from a previous study by other groups [25], we examined whether Frizzled-7 (FZD7), a key receptor in Wnt/β-catenin pathway activation, would possibly be a downstream target of Sox9. FZD7 is overexpressed in human HCC [26] and activates the Wnt/β-catenin pathway [27, 28]. We found by qPCR and Western blotting FZD7 expression was suppressed in Sox9 knockdown clones compared with NTC. The reversed results were observed upon transient Sox9 overexpression (Figure 6A). Using the web-based prediction program ConSite for transcription factor binding analysis, several putative Sox9 binding sites were found on FZD7. ChIP assays results showed that Sox9 binds to FZD7 at R1, R2 and R3 regions. Among these three regions, binding at R2 showed the highest fold of enrichment (17.52-fold), while R1 with 2.84-fold and R3 with 8.40-fold when compared with normal rabbit IgG control (Figure 6B). To further confirm the direct binding of Sox9 on these regions, we examined the FZD7 promoter activity in Sox9 expression-altered HCC cells using luciferase reporter assay. Significant decrease of signals was detected in all three regions in Sox9-silenced Huh7. Similar fold change of luciferase signals were detected at R1 (0.65- and 0.71-fold) and R3 (0.66- and 0.69-fold) in both knockdown clones #386 ad #388. The signals were significantly enhanced after Sox9-overexpression in SMMC-7721, with 3.12- and 3.14-fold at R1 and R3, respectively. The binding at R2 was most significantly suppressed by 0.52-fold in shSox9#386, while largely upregulated to 4.73-fold in Sox9-overexpressing SMMC-7721 when compared with the respective control groups, which agreed with our findings in ChIP assay of highest fold of enrichment detected at R2 (Figure 6C). This transcriptional relationship was further supported by a positive correlation of mRNA levels of FZD7 and Sox9 in our clinical HCC cohort (Figure 6D). These findings suggest that Sox9 activates Wnt/β-catenin signaling through transcriptional regulation of FZD7 (followed by phosphorylation of GSK3β and the downstream events).

## DISCUSSION

In this study we demonstrated by expression analysis, together with *in vitro* and *in vivo* experiments, that Sox9 endows stemness features in human HCC. Sox9 expression in HCC cells was associated with that of stemness markers. Silencing of Sox9 inhibited HCC proliferation, migration and invasion abilities and suppressed tumorsphere formation as well as side population. We also demonstrated that Sox9 confers chemoresistance in HCC. *In vivo*, silencing of Sox9 suppressed tumorigenicity and lung metastasis. We also found that Sox9 endows these properties in HCC through the canonical Wnt pathway. In addition, we demonstrated that FZD7, a key receptor of the pathway, is a direct transcriptional target of Sox9 in HCC. The summary of findings in this study is illustrated in Supplementary Figure S5.

Sox9 in recent years has been identified as a stem/progenitor cell marker for the liver and the pancreas [2]. It is suggested that Sox9 carries a progenitor cell function in some physiological and pathological processes. In our clinical cohort Sox9 is overexpressed in HCC tissues and associated with higher tumor grade, venous invasion, advanced tumor stage and poorer overall survival. These findings are similar to those reported by Guo et al. [13]. In the current study we additionally provided substantiating data through an in-depth characterization of Sox9 in HCC with an emphasis on stemness features. Our findings consolidated the role of Sox9 on conferring stemness properties in human HCC. A vast majority of HCC arises from a background of chronic liver disease (hepatitis virus infection, fatty liver disease, etc.) and the ensuing cirrhosis. Proliferation of Sox9+ progenitor cells during liver injury may be implicated in multi-step hepatocarcinogenesis. Sox9+ cells in HCC are possibly derived from Sox9+ liver progenitor cells.

The canonical Wnt pathway is crucial in hepatocarcinogenesis [29]. In this regard, β-catenin mutation occurs in up to 40% of HCC [30] and it was well noted that a proportion of tumors showing accumulation of β-catenin protein do not carry β-catenin mutation [31]. In other words, heterogeneous regulatory mechanisms are involved in the activation of β-catenin. In this study, we showed that Sox9 confers HCC stemness properties through Wnt/β-catenin signaling. Moreover, we proved that Sox9, as a transcription factor, drives the transcription of FZD7, a key receptor of the canonical Wnt pathway in HCC. These findings enrich our understanding on Wnt/β-catenin signaling in hepatocarcinogenesis, in which Sox9 is an important and indispensible player. Recent works suggested that FZD7 emerges as a crucial regulator for stemness features in both human embryonic stem cells and cancer stem cells [32, 33]. Activation in the Wnt/β-catenin pathway through FZD7 may carry specific implications in the context of cancer stemness. The findings in our study suggest that Sox9/FZD7/Wnt signaling is implicated in endowing stemness properties of HCC.

In this study we identified the downstream target and signaling pathway of Sox9 in HCC. What remains to be a question is the regulatory mechanism of Sox9 expression in this cancer. In breast cancer, Wnt increases Sox9 expression [22]. In lung and esophageal cancers Sox9 is activated by Notch pathway [9, 34] and in the esophagus, Sox9 is also a YAP1 target [35]. While in HCC, suppression of Sox9 by miRNA-101 and miRNA-145 inhibits tumor proliferation, migration and invasion [36]. There is also evidence that activation of Notch and β-catenin pathways increases Sox9+ cells in HCC [37, 38]. Apart from that, Sox9 expression is upregulated by hypoxia in ovarian cancer [39]. Hypoxia is known to be associated with cancer stemness [40]. In view of this we retrieved our RNA sequencing data on hypoxia (1% O_2_) treated HCC cells and compared that under normoxic condition (20% O_2_). On the whole, we observed no drastic upregulation of Sox9 RNA copy number under hypoxic condition. The fold change in PLC/PRF/5, SMMC-7721, Hep3B, and MHCC-97L is 3.330, 1.856, 0.796 and 1.796 respectively. Thus hypoxia may not be a sufficient mechanism for Sox9 upregulation in HCC. Lines of evidences have shown promoter site hypomethylation is an epigenetic hallmark for genes conferring CSC properties [41]. Moreover, epigenetic regulation of Sox9 expression by DNA methylation was observed in pancreatic cancer [42]. In this connection, DNA methylation of Sox9 promoter region possibly determines Sox9 expression levels in liver tissues.

Our findings suggest that Sox9 could serve as a molecular target with prognostic significance in HCC, as well as a predictive marker for chemotherapy response. Being remarkably crucial in regulating multiple stemness properties including metastasis, Sox9 is possibly a critical therapeutic target for HCC so as to improve clinical outcome and patient survival. Use of specific inhibitors is one of the potential approaches to suppress the oncogenic functions Sox9 in HCC and to augment treatment effects of current chemotherapeutic agents. Further dissection of the epigenetic regulation of Sox9 expression in this cancer may also provide insights on the therapeutic perspective.

## MATERIALS AND METHODS

### Human HCC samples

Human tissues in the HCC cohort were obtained from patients with liver resection at Queen Mary Hospital, Hong Kong and randomly selected for the current study. All specimens collected were either snap-frozen in liquid nitrogen and stored at −80°C, or fixed in 10% formalin for paraffin embedding. Frozen sections from tissue samples were cut and stained for histological examination to ensure homogenous cell population. Use of human specimens was approved by the Institutional Review Board of the University of Hong Kong/Hospital Authority Hong Kong West Cluster (Reference Number UW 14-049).

### Cell line models

Human HCC cell lines BEL-7402, SMMC-7721, PLC/PRF/5, Huh7, Hep3B, HepG2, MHCC-97L, MHCC-97H and immortalized normal liver cell line LO2 were used. BEL-7402 and SMMC-7721 were obtained from Shanghai Institute of Cell Biology. PLC/PRF/5, Hep3B and HepG2 were purchased from American Type Culture Collection (Manassas, VA). MHCC-97L and MHCC-97H were gifts from Professor Z.Y. Tang at Fudan University. Huh7 was from Dr. H. Nakabayashi at Hokkaido University School of Medicine. LO2 was from Dr. J.R. Gu at the Shanghai Cancer Institute.

### Sox9 knockdown and overexpressing HCC cells

To establish Sox9 knockdown clones, recombinant lentivirus was generated by co-transfecting 293FT cells with shSox9 from Sigma-Aldrich Mission^®^ shRNA bacterial glycerol stock, Clone IDs: TRCN0000020386 (shSox9#386) and TRCN0000020388 (shSox9#388) or non-target control (NTC) plasmids and packaging plasmid mix (System Biosciences, Mountain View, CA). Viral supernatant was collected and used to infect Huh7 and Hep3B. The infected cells were selected with puromycin (Sigma-Aldrich). For overexpression study, either Sox9 expression construct (SC321884: NM_000346, human cDNA clone) or empty vector (Origene Technologies, Rockville, MD) were transfected into SMMC-7721 using Lipofectamine^®^ 2000.

### Antibodies

Anti-Sox9 antibody was purchased from Millipore (AB5535, Billerica, MA). Anti-β-catenin and glycogen synthase kinase 3β (GSK3β) antibodies were obtained from BD Transduction Laboratories (#610153 & #610201, San Jose, CA). Anti-Oct4, Sox2 and FZD7 antibodies were purchased from Abcam (ab19857, ab97959 & ab51049, Cambridge, UK). Anti-E-cadherin, vimentin and phospho-GSK3β (Ser9) antibodies were obtained from Cell Signaling Technology (#3195, #5741 & #9336, Beverly, MA, USA). Anti-β-actin antibody was purchased from Sigma-Aldrich (A5316, St. Louis, MO, USA). Anti-CD24 (ab30350, Abcam), E-cadherin (#610181, BD Transduction Laboratories) and vimentin antibodies (M0725, Dako, Glostrup, Denmark) were used for immunofluorescent staining. Anti-CK19 (ab52625, Abcam), anti-AFP (A0008, Dako) and anti-EpCAM (M0804, Dako) were used for immunohistochemistry.

### Immunohistochemistry (IHC)

Immunohistochemical staining for Sox9, CK19, AFP and EpCAM were performed on formalin-fixed, paraffin-embedded sections using labeled horseradish peroxidase (HRP) method. After heat antigen retrieval with Tris-EDTA buffer (Sox9, CK19 & AFP) or Protease K enzyme antigen retrieval (EpCAM), endogenous peroxidase activities were quenched by 3% H_2_O_2_. The sections were immersed in serum free-protein block solution (Dako) and incubated with anti-Sox9 (dilution 1:1000), anti-CK19 (dilution 1:1000), anti-AFP (dilution 1:6000) and anti-EpCAM (dilution 1:100) at 4°C overnight. The sections were thoroughly washed and incubated with Envision^™^ HRP-conjugated secondary antibody (Dako). Positive signals were visualized using 3,3′-diaminobenzidine (Dako). Nuclei were counterstained with hematoxylin.

### Western blotting

Whole cell lysates from cell lines were extracted using RIPA with protease and phosphatase inhibitors (Roche, Mannheim, Germany). The concentration of protein lysates was determined by Bradford assay (BioRad, Hercules, CA). Protein were separated by SDS-polyacrylamide gel electrophoresis (SDS-PAGE), transferred to polyvinylidene difluoride membrane (Millipore), blocked and incubated with primary antibodies. HRP-conjugated anti-rabbit or anti-mouse IgG were used where appropriate (GE Healthcare Life Sciences, Piscataway, NJ). The signals were visualized using enhanced chemiluminescence method.

### RNA extraction and quantitative reverse-transcription polymerase chain reaction (qPCR)

Total RNA was extracted using Trizol reagent (Life Technologies) according to manufacturer's protocol. One microgram of RNA was used for cDNA synthesis using GeneAmp RNA PCR Kit (Applied Biosystems, Life Technologies). Expression of Sox9 of clinical samples was examined using TaqMan^®^ Assay target probes (Assay ID: Hs01001343_g1), normalized using 18s rRNA (#4319413E, Applied Biosystems, Life Technologies). Expression of chemoresistance-related genes, stemness-associated genes, c-myc and axin2 was examined by Power SYBR^®^ Green PCR Master Mix (#4367659, Applied Biosystems, Life Technologies) using the primers indicated in Supplementary Table S3 with Applied Biosystems 7900HT Fast Real-Time PCR System.

### Immunofluorescent (IF) staining

Cells of interest grown on chamber slides were fixed and permeabilized. Following blocking with 10% goat and donkey serum (G9023 and D9663, Sigma-Aldrich), they were incubated with primary antibodies overnight at 4°C. The slides were washed and incubated with Alexa-Fluor^®^-488 donkey anti-mouse IgG and Alexa-Fluor^®^-568 goat anti-rabbit IgG (A21202 and A11011, Molecular Probes^®^, Invitrogen) together with 4′,6-Diamidino-2-Phenylindole (DAPI) (D-1306, Molecular Probes^®^) for nucleus staining. All images were captured using Zeiss LSM700 inverted confocal microscope (Gottingen, Germany) at Faculty Core Facility, Li Ka Shing Faculty of Medicine, the University of Hong Kong.

### Cell proliferation assay

Proliferation assay was performed using cell counting method. HCC cells were seeded at 5 × 10^3^ per well into 24-well plate and counted in triplicates each day for seven days.

### Cell invasion and migration transwell assays

Migration assays were performed using polycarbonate membrane transwell inserts with pore size of 8.0 μm. Invasion assays were performed using Matrigel (BD Biosciences) coated transwell inserts. Cells in serum free medium were seeded in upper chamber while the lower chamber was filled with medium supplemented with 10% FBS as chemoattractant. The transwell membranes were harvested after 24 hours, fixed and stained with 1% crystal violet. The membranes were cleaned, air-dried and mounted. The number of cells migrated or invaded was counted in 5 random fields.

### Tumorsphere formation assay

Five hundreds HCC cells were seeded to 24-well plates coated with polyHEMA (Sigma Aldrich). Cells were grown in DMEM/F12 medium (Invitrogen, Life Technologies) supplemented with 4 μg/mL insulin (Sigma-Aldrich), 20 ng/mL EGF (Sigma-Aldrich), B27 (Invitrogen, Life Technologies) and 20 ng/mL basic FGF (Invitrogen, Life Technologies) for either 10 days (Huh7) or 15 days (Hep3B). For the secondary propagation of primary tumorspheres, primary tumorspheres were collected by centrifugation. The tumorspheres collected were washed with PBS, subsequently subjected to centrifugation and dissociated to single cell suspension using trypsin. After inactivating the trypsin, the cells were resuspended with DMEM/F12 medium with the above supplements and cultured in polyHEMA-coated plates.

### Side population analysis

HCC cells were detached and suspended at 1 × 10^6^ cells/mL in DMEM medium supplemented with 2% FBS and 10 mM HEPES (pH 7.4). These cells were incubated at 37°C for 90 minutes with 5 μg/mL Hoechst 33342 (Sigma-Aldrich), either alone or in the presence of 5 μM of ABC-transporter inhibitor Reserpine (Sigma-Aldrich). After incubation, the cells were co-stained with 5 μg/mL propidium iodide for dead cell exclusion (BD Pharmingen^™^). The cells were rinsed with ice-cold PBS supplemented with 2% FBS and 10 mM HEPES (pH 7.4) buffer. Cell analysis and data acquisition was performed with FACS Aria SORP cytometer (BD Biosciences) using DIVA software.

### Annexin V-apoptosis assay

HCC cells were treated with either doxorubicin or cisplatin in serum free condition for 24 hours. Apoptosis assays were performed using Annexin V-FITC Apoptosis Detection Kit I (556547, BD Pharmingen™) according to manufacturer's protocol. The stained cells were subjected to analyses using either FACSCantoII Analyzer or FACSCalibur flow cytometer and CellQuest software (BD BioSciences).

### β-catenin TCF binding assay

The effect of Sox9 towards β-catenin activity was examined using luciferase reporter assay of TCF/LEF-dependent transcription. Either 1.0 μg Firefly luciferase pSuper8XTOPflash or pSuper8XFOPflash constructs (gifts from Dr. Moon R, University of Washington, USA) together with 0.2 μg Renilla luciferase pRL-CMV construct (Promega, Madison, WI, USA) were transfected into HCC cells using Lipofectamine^®^ 2000. Luciferase activities were assayed using Dual-Luciferase^®^ Reporter Assay System (E1910, Promega) according to manufacturer's protocol.

### *In vivo* subcutaneous xenograft inoculation

The study protocol was approved by the Committee of the Use of Live Animals in Teaching and Research at the University of Hong Kong. One million of either NTC or shSox9#386 Huh7 cells were injected subcutaneously at both sides of posterior flanks of 4- to 6-week old male non-obese diabetic (NOD)/SCID mice (NOD.CB17-*Prkdc*^scid^/J). For limited dilution assay and β-catenin overexpression rescue-experiment, 5 × 10^3^, 1 × 10^4^ or 5 × 10^4^ cells were injected subcutaneously into the mice. Tumor volume was calculated by the formula length × width^2^ × 0.5. Mice were sacrificed at 4 weeks (1 × 10^6^ cells), 70 days (5 × 10^3^ cells), 75 or 60 days (1 × 10^4^ cells) or 57 days (5 × 10^4^ cells) and tumor bulks were harvested. All experiments were performed according to the Animals (Control of experiments) Ordinance (Hong Kong).

### *In vivo* tail vein injection

One million of either NTC or shSox9#386 luciferase-labeled BEL-7402 cells were injected into the tail vein of 6-week old NOD/SCID mice. *Ex vivo* lung metastases were recorded using IVIS 100 Imaging System (Xenogen, Hopkinton, MA) at week 12 after anesthetizing and injecting 100 mg/kg D-luciferin intraperitoneally into the mice. Lung tissues were harvested for histological analyses.

### Chromatin immunoprecipitation (ChIP) assay

ChIP assay was carried out according to the manufacturer's protocol (ChIP assay Kit, Millipore). The precleared lysates were immunoprecipitated with Sox9 antibody and normal rabbit IgG (sc-2027, Santa Cruz). The pull-down purified DNA was quantified using qPCR, with the primers indicated in Supplementary Table S4.

### Luciferase reporter assay

The regions of interest of Frizzled-7 were amplified using the primers indicated in Supplementary Table S5 and cloned into pGL3-Basic construct (Promega). Cloned construct together with pRL-CMV were transfected into the cells of interest. The cells were harvested after 24 hours and luciferase activities were measured using Dual-Luciferase^®^ Reporter Assay System.

### Statistical analysis

Statistical analysis for clinicopathological correlation was performed using SPSS 19 for Windows (SPSS, Inc., Chicago, IL). Fisher's exact test was used for categorical data. Survival curves of Sox9 overexpression (T/NT at 4-fold cutoff) were determined by the Kaplan-Meier method, and the statistical difference between two groups was evaluated by the Log-rank test. All experimental data were analyzed using *t* test or Mann-Whitney's *U* test wherever appropriate (SigmaPlot 10.0 and SigmaStat 3.1, Jandel Scientific, San Rafael, CA; or GraphPad Prism 5.0, La Jolla, CA, USA). Statistical significance was defined by *p* < 0.05.

## SUPPLEMENTARY MATERIALS TABLES AND FIGURES



## References

[1] Lefebvre V, Dumitriu B, Penzo-Mendez A, Han Y, Pallavi B (2007). Control of cell fate and differentiation by Sry-related high-mobility-group box (Sox) transcription factors. Int J Biochem Cell Biol.

[2] Furuyama K, Kawaguchi Y, Akiyama H, Horiguchi M, Kodama S, Kuhara T, Hosokawa S, Elbahrawy A, Soeda T, Koizumi M, Masui T, Kawaguchi M, Takaori K (2011). Continuous cell supply from a Sox9-expressing progenitor zone in adult liver, exocrine pancreas and intestine. Nat Genet.

[3] Yimlamai D, Christodoulou C, Galli GG, Yanger K, Pepe-Mooney B, Gurung B, Shrestha K, Cahan P, Stanger BZ, Camargo FD (2014). Hippo pathway activity influences liver cell fate. Cell.

[4] Zhong WD, Qin GQ, Dai QS, Han ZD, Chen SM, Ling XH, Fu X, Cai C, Chen JH, Chen XB, Lin ZY, Deng YH, Wu SL (2012). SOXs in human prostate cancer: implication as progression and prognosis factors. BMC Cancer.

[5] Zhou CH, Ye LP, Ye SX, Li Y, Zhang XY, Xu XY, Gong LY (2012). Clinical significance of SOX9 in human non-small cell lung cancer progression and overall patient survival. J Exp Clin Cancer Res.

[6] Lu B, Fang Y, Xu J, Wang L, Xu F, Xu E, Huang Q, Lai M (2008). Analysis of SOX9 expression in colorectal cancer. Am J Clin Pathol.

[7] Jiang SS, Fang WT, Hou YH, Huang SF, Yen BL, Chang JL, Li SM, Liu HP, Liu YL, Huang CT, Li YW, Jang TH, Chan SH (2010). Upregulation of SOX9 in lung adenocarcinoma and its involvement in the regulation of cell growth and tumorigenicity. Clin Cancer Res.

[8] Huang Z, Hurley PJ, Simons BW, Marchionni L, Berman DM, Ross AE, Schaeffer EM (2012). Sox9 is required for prostate development and prostate cancer initiation. Oncotarget.

[9] Song S, Maru DM, Ajani JA, Chan CH, Honjo S, Lin HK, Correa A, Hofstetter WL, Davila M, Stroehlein J, Mishra L (2013). Loss of TGF-beta Adaptor beta2SP Activates Notch Signaling and SOX9 Expression in Esophageal Adenocarcinoma. Cancer Res.

[10] Matheu A, Collado M, Wise C, Manterola L, Cekaite L, Tye AJ, Canamero M, Bujanda L, Schedl A, Cheah KS, Skotheim RI, Lothe RA, Lopez de Munain A (2012). Oncogenicity of the developmental transcription factor Sox9. Cancer Res.

[11] Guo W, Keckesova Z, Donaher JL, Shibue T, Tischler V, Reinhardt F, Itzkovitz S, Noske A, Zurrer-Hardi U, Bell G, Tam WL, Mani SA, van Oudenaarden A (2012). Slug and Sox9 cooperatively determine the mammary stem cell state. Cell.

[12] Grimont A, Pinho AV, Cowley MJ, Augereau C, Mawson A, Giry-Laterriere M, Van den Steen G, Waddell N, Pajic M, Sempoux C, Wu J, Grimmond SM, Biankin AV (2015). SOX9 regulates ERBB signalling in pancreatic cancer development. Gut.

[13] Guo X, Xiong L, Sun T, Peng R, Zou L, Zhu H, Zhang J, Li H, Zhao J (2012). Expression features of SOX9 associate with tumor progression and poor prognosis of hepatocellular carcinoma. Diagn Pathol.

[14] Malanchi I, Santamaria-Martinez A, Susanto E, Peng H, Lehr HA, Delaloye JF, Huelsken J (2012). Interactions between cancer stem cells and their niche govern metastatic colonization. Nature.

[15] Jordan CT, Guzman ML, Noble M (2006). Cancer stem cells. N Engl J Med.

[16] Yamashita T, Wang XW (2013). Cancer stem cells in the development of liver cancer. J Clin Invest.

[17] Lee TK, Castilho A, Cheung VC, Tang KH, Ma S, Ng IO (2011). CD24(+) liver tumor-initiating cells drive self-renewal and tumor initiation through STAT3-mediated NANOG regulation. Cell Stem Cell.

[18] Lee TK, Cheung VC, Ng IO (2013). Liver tumor-initiating cells as a therapeutic target for hepatocellular carcinoma. Cancer Lett.

[19] Kim H, Choi GH, Na DC, Ahn EY, Kim GI, Lee JE, Cho JY, Yoo JE, Choi JS, Park YN (2011). Human hepatocellular carcinomas with “Stemness”-related marker expression: keratin 19 expression and a poor prognosis. Hepatology.

[20] Akiyama H, Kamitani T, Yang X, Kandyil R, Bridgewater LC, Fellous M, Mori-Akiyama Y, de Crombrugghe B (2005). The transcription factor Sox9 is degraded by the ubiquitin-proteasome system and stabilized by a mutation in a ubiquitin-target site. Matrix Biol.

[21] McDonald E, Li J, Krishnamurthy M, Fellows GF, Goodyer CG, Wang R (2012). SOX9 regulates endocrine cell differentiation during human fetal pancreas development. Int J Biochem Cell Biol.

[22] Wang H, He L, Ma F, Regan MM, Balk SP, Richardson AL, Yuan X (2013). SOX9 regulates low density lipoprotein receptor-related protein 6 (LRP6) and T-cell factor 4 (TCF4) expression and Wnt/beta-catenin activation in breast cancer. J Biol Chem.

[23] Liu H, Liu Z, Jiang B, Peng R, Ma Z, Lu J (2015). SOX9 Overexpression Promotes Glioma Metastasis via Wnt/beta-Catenin Signaling. Cell Biochem Biophys.

[24] Morin PJ, Sparks AB, Korinek V, Barker N, Clevers H, Vogelstein B, Kinzler KW (1997). Activation of beta-catenin-Tcf signaling in colon cancer by mutations in beta-catenin or APC. Science.

[25] Kadaja M, Keyes BE, Lin M, Pasolli HA, Genander M, Polak L, Stokes N, Zheng D, Fuchs E (2014). SOX9: a stem cell transcriptional regulator of secreted niche signaling factors. Genes Dev.

[26] Bengochea A, de Souza MM, Lefrancois L, Le Roux E, Galy O, Chemin I, Kim M, Wands JR, Trepo C, Hainaut P, Scoazec JY, Vitvitski L, Merle P (2008). Common dysregulation of Wnt/Frizzled receptor elements in human hepatocellular carcinoma. Br J Cancer.

[27] Merle P, Kim M, Herrmann M, Gupte A, Lefrancois L, Califano S, Trepo C, Tanaka S, Vitvitski L, de la Monte S, Wands JR (2005). Oncogenic role of the frizzled-7/beta-catenin pathway in hepatocellular carcinoma. J Hepatol.

[28] Kim M, Lee HC, Tsedensodnom O, Hartley R, Lim YS, Yu E, Merle P, Wands JR (2008). Functional interaction between Wnt3 and Frizzled-7 leads to activation of the Wnt/beta-catenin signaling pathway in hepatocellular carcinoma cells. J Hepatol.

[29] Singh Monga SP (2015). beta-Catenin Signaling and Roles in Liver Homeostasis, Injury And Tumorigenesis. Gastroenterology.

[30] Nejak-Bowen KN, Monga SP (2011). Beta-catenin signaling, liver regeneration and hepatocellular cancer: sorting the good from the bad. Semin Cancer Biol.

[31] Wong CM, Fan ST, Ng IO (2001). beta-Catenin mutation and overexpression in hepatocellular carcinoma: clinicopathologic and prognostic significance. Cancer.

[32] King TD, Zhang W, Suto MJ, Li Y (2012). Frizzled7 as an emerging target for cancer therapy. Cell Signal.

[33] Flanagan DJ, Phesse TJ, Barker N, Schwab RH, Amin N, Malaterre J, Stange DE, Nowell CJ, Currie SA, Saw JT, Beuchert E, Ramsay RG, Sansom OJ (2015). Frizzled7 Functions as a Wnt Receptor in Intestinal Epithelial Lgr5 Stem Cells. Stem Cell Reports.

[34] Capaccione KM, Hong X, Morgan KM, Liu W, Bishop JM, Liu L, Markert E, Deen M, Minerowicz C, Bertino JR, Allen T, Pine SR (2014). Sox9 mediates Notch1-induced mesenchymal features in lung adenocarcinoma. Oncotarget.

[35] Song S, Ajani JA, Honjo S, Maru DM, Chen Q, Scott AW, Heallen TR, Xiao L, Hofstetter WL, Weston B, Lee JH, Wadhwa R, Sudo K (2014). Hippo Coactivator YAP1 Upregulates SOX9 and Endows Esophageal Cancer Cells with Stem-like Properties. Cancer Res.

[36] Zhang Y, Guo X, Xiong L, Kong X, Xu Y, Liu C, Zou L, Li Z, Zhao J, Lin N (2012). MicroRNA-101 suppresses SOX9-dependent tumorigenicity and promotes favorable prognosis of human hepatocellular carcinoma. FEBS Lett.

[37] Villanueva A, Alsinet C, Yanger K, Hoshida Y, Zong Y, Toffanin S, Rodriguez-Carunchio L, Sole M, Thung S, Stanger BZ, Llovet JM (2012). Notch signaling is activated in human hepatocellular carcinoma and induces tumor formation in mice. Gastroenterology.

[38] Mokkapati S, Niopek K, Huang L, Cunniff KJ, Ruteshouser EC, deCaestecker M, Finegold MJ, Huff V (2014). beta-catenin activation in a novel liver progenitor cell type is sufficient to cause hepatocellular carcinoma and hepatoblastoma. Cancer Res.

[39] Raspaglio G, Petrillo M, Martinelli E, Li Puma DD, Mariani M, De Donato M, Filippetti F, Mozzetti S, Prislei S, Zannoni GF, Scambia G, Ferlini C (2014). Sox9 and Hif-2alpha regulate TUBB3 gene expression and affect ovarian cancer aggressiveness. Gene.

[40] Yun Z, Lin Q (2014). Hypoxia and regulation of cancer cell stemness. Adv Exp Med Biol.

[41] Hernandez-Vargas H, Sincic N, Ouzounova M, Herceg Z (2009). Epigenetic signatures in stem cells and cancer stem cells. Epigenomics.

[42] Sun L, Mathews LA, Cabarcas SM, Zhang X, Yang A, Zhang Y, Young MR, Klarmann KD, Keller JR, Farrar WL (2013). Epigenetic regulation of SOX9 by the NF-kappaB signaling pathway in pancreatic cancer stem cells. Stem Cells.

