# Supplementary material for: Sox9 confers stemness properties in hepatocellular carcinoma through Frizzled-7 mediated Wnt/β-catenin signaling
Source: Oncotarget. 2016 Apr 19;7(20):29371–86. doi: 10.18632/oncotarget.8835 (PMC5045402; doi:10.18632/oncotarget.8835)
Supplement: Supplementary file 1 [file oncotarget-07-29371-s001.pdf]

Sox9 confers stemness properties in hepatocellular carcinoma through Frizzled–7 mediated Wnt/β–catenin signaling

Supplementary Materials

Supplementary Table S1: Correlation of Sox9 mRNA and protein levels in HCC clinical samples (*n* = 67, *p* = 0.0008, Fisher's exact test)

		Sox9 protein[@]			
		Overexpressed	Not overexpressed	Total	*P* value (Fisher's Exact Test)
Sox9 mRNA	T/NT ≥ 4–fold	30	1	31	0.0008***
	T/NT < 4–fold	23	13	36	

[@] Positive staining by IHC in tumor cells (compared with corresponding non–tumor cells).
T: tumor; NT: corresponding non–tumorous liver tissue.

Supplementary Table S2: Immunohistochemical expressions of Sox9, CK19, AFP and EpCAM in clinical HCC cohort

Case number	Sox 9[#]	CK19	AFP	EpCAM
1	H	–	+	–
2	–	–		
3	–	–		
4	–	–		
5	H	+	–	+
6	–	–		
7	–	–		
8	H	–	+	–
9	–	–		
10	L	–	–	–
11	H	–	+	+
12	H	–	–	–
13	L	–	–	–
14	H	+	+	+
15	–	–		
16	–	–		
17	–	–		
18	L	–	–	–
19	H	–	–	–
20	H	–	–	+
21	H	+	+	–
22	L	–	–	–
23	H	+	+	+

24	H	+	+	+
25	L	–	+	–
26	H	–	+	–
27	H	–	–	–
28	L	–	+	
29	H	–	+	+
30	L	–	–	–
31	H	–	+	–
32	L	+	–	–
33	L	–	–	–
34	L	–	–	+
35	–	–		
36	L	–	+	+
37	H	+	+	+
38	H	–	+	–
39	H	–	+	+
40	H	–	–	+
41	H	–	–	–
42	H	+	–	+
43	L	–	–	–
44	–	–		
45	–	–		
46	H	+	+	–
47	L	+	–	+
48	H	+	–	+
49	L	–	–	–
50	H	–	–	+
51	H	–	–	–
52	H	+	–	+
53	H	–	–	–
54	H	–	–	–
55	H	+	+	–
56	H	–	–	–
57	L	+	–	+
58	L	–	–	–
59	H	+	–	–
60	L	–	+	+
61	H	+	–	–
62	–	–		
63	–	–		
64	H	+	+	+
65	H	–	+	+
66	H	–	–	–
67	H	–	+	+

[#] Staining in tumor cells—H (High): > 33%; L (Low): 1–33%; – (Negative):0%.

Supplementary Table S3: Primer sequences for qPCR

Gene		Primer Sequences (5′ → 3′)
ABCB1	Forward	AAATTGGCTTGACAAGTTGTATATGG
	Reverse	CACCAGCATCATGAGAGGAAGTC
ABCC6	Forward	TTGGATTCGCCCTCATAGTC
	Reverse	GGTAGCTGGCAAGACAAAGC
ABCG2	Forward	TCATCAGCCTCGATATTCCATCT
	Reverse	GGCCCGTGGAACATAAGTCTT
Axin2	Forward	CAGCGAGTATTACTGCTACTCGAAA
	Reverse	TTTTTTGTGCTTTGGGCACTATG
BMI–1	Forward	TGGAGAAGGAATGGTCCACTTC
	Reverse	GTGAGGAAACTGTGGATGAGGA
c–myc	Forward	CGTCCTCGGATTCTCTGCTC
	Reverse	GCTGGTGCATTTTCGGTTGT
CD24	Forward	TGAAGAACATGTGAGAGGTTTGAC
	Reverse	GAAAACTGAATCTCCATTCCACAA
CD133	Forward	TGGATGCAGAACTTGACAACGT
	Reverse	ATACCTGCTACGACAGTCGTGGT
FZD7	Forward	CGCGGCCGCTCCGCTTTC
	Reverse	GCGCTCGCACAGAGAACGACA
Nanog	Forward	AATACCTCAGCCTCCAGCAGATG
	Reverse	TGCGTCACACCATTGCTATTCTTC
Nestin	Forward	CTGCGGGCTACTGAAAAGTT
	Reverse	AGGCTGAGGGACATCTTGAG
Notch 1	Forward	CCTGAGGGCTTCAAAGTGTC
	Reverse	CGGAACTTCTTGGTCTCCAG
Oct4	Forward	CTTGCTGCAGAAGTGGGTGGAGGAA
	Reverse	CTGCAGTGTGGGTTTCGGGCA
Sox2	Forward	AAATGGGAGGGGTGCAAAAGAGGAG
	Reverse	CAGCTGTCATTTGCTGTGGGTGATG
Sox9	Forward	AGCGAACGCACATCAAGAC
	Reverse	CTGTAGGCGATCTGTGGGG

Supplementary Table S4: Primer sequences for ChIP assay

FZD7 ChIP		Primer Sequence (5′ → 3′)
R1	Forward	GGTAGCCATCGTCCGAGAAG
	Reverse	CTACCGCTTCCTGGGTGAG
R2	Forward	AACTTGTTCATGAGCGCCTC
	Reverse	GCCTACAACCAGACCATCCT
R3	Forward	TCTGAGTGCACCAGGAAGAG
	Reverse	CCCATTCACAAACCACTCCG
R4	Forward	TCCTGATATTCCTGGCAGCC
	Reverse	TCAAAACACAAAGCGGGGTT

Supplementary Table S5: Primer sequences for FZD7 luciferase reporter assay

FZD7		Primer Sequence (5′ → 3′)
R1	Forward	**ATCGCTAGC**ATGAAGTAGCAGCCCGACAG
	Reverse	**CTCAAGCTT**CCACTGCCTACCCTACCG
R2	Forward	**ATCGCTAGC**AGCCGAACTTGTTCATGAGC
	Reverse	**CTCAAGCTT**GTACCACGGAGAGAAGGGC
R3	Forward	**ATCGCTAGC**TGCACCAGGAAGAGGAACAA
	Reverse	**CTCAAGCTT**GTCGCCAGAAGAAGCGTC

Restriction enzyme digested sites added in the primer sequences.
GCTAGC: NheI.
AAGCTT: HindIII.

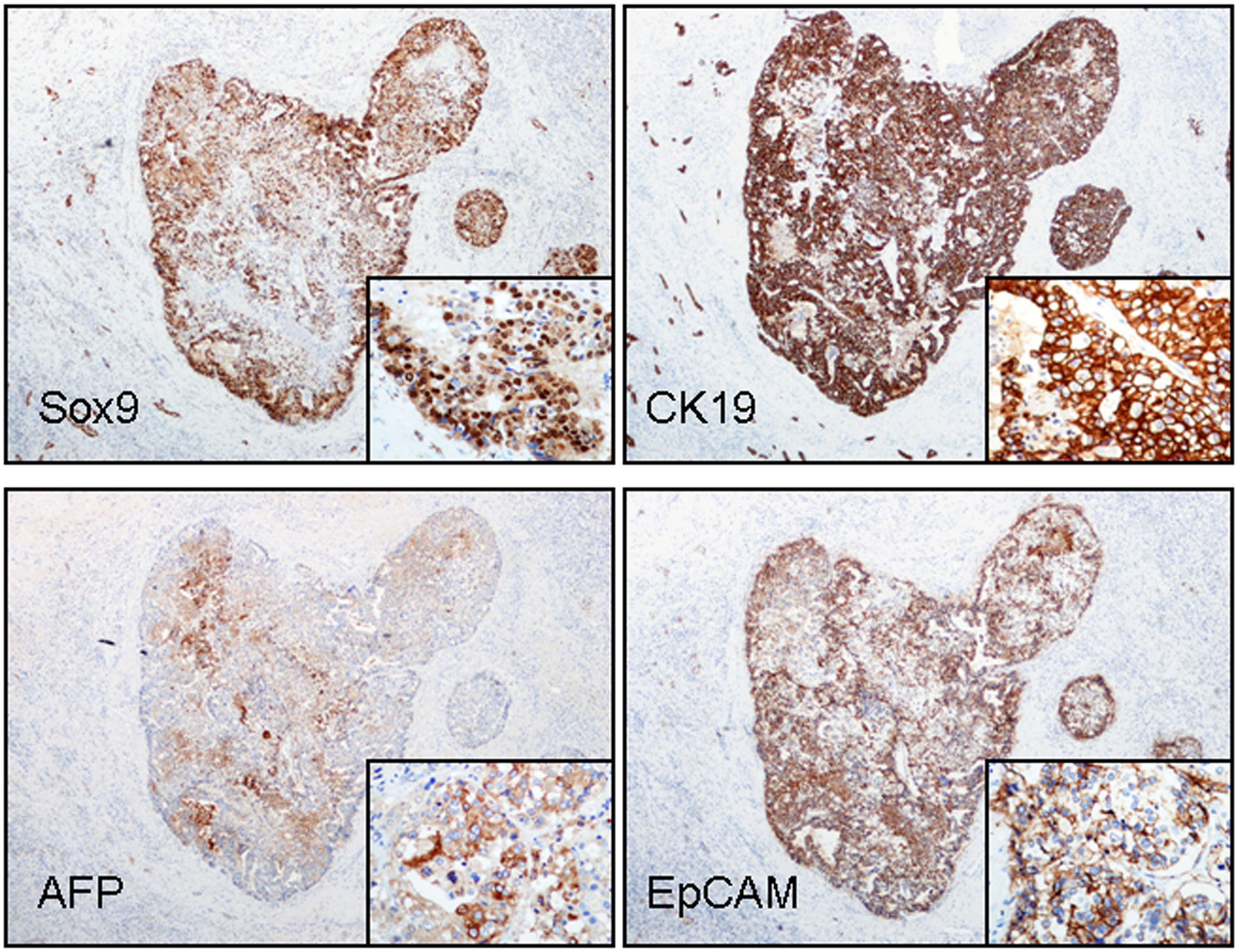

Supplementary Figure S1: Representative images for immunohistochemical staining of Sox9 and other stemness markers CK19, AFP and EpCAM in a clinical HCC sample (40×); higher magnifications (400×) shown in insets.

No. of cells	Tumor incidence		Latency (days)	
	NTC	shSox9#386	NTC	shSox9#386
$1X10^4$	6/6 (100%)	1/6 (16.7%)	41.7 ± 1.7	66
$5X10^4$	5/5 (100%)	1/5 (20%)	39 ± 2.4	57

Supplementary Figure S2: Silencing of Sox9 reduced tumor incidence and prolonged tumor latency *in vivo*. Upon knockdown of Sox9, lower tumor incidence and longer tumor latency period were observed with both cell numbers injected.

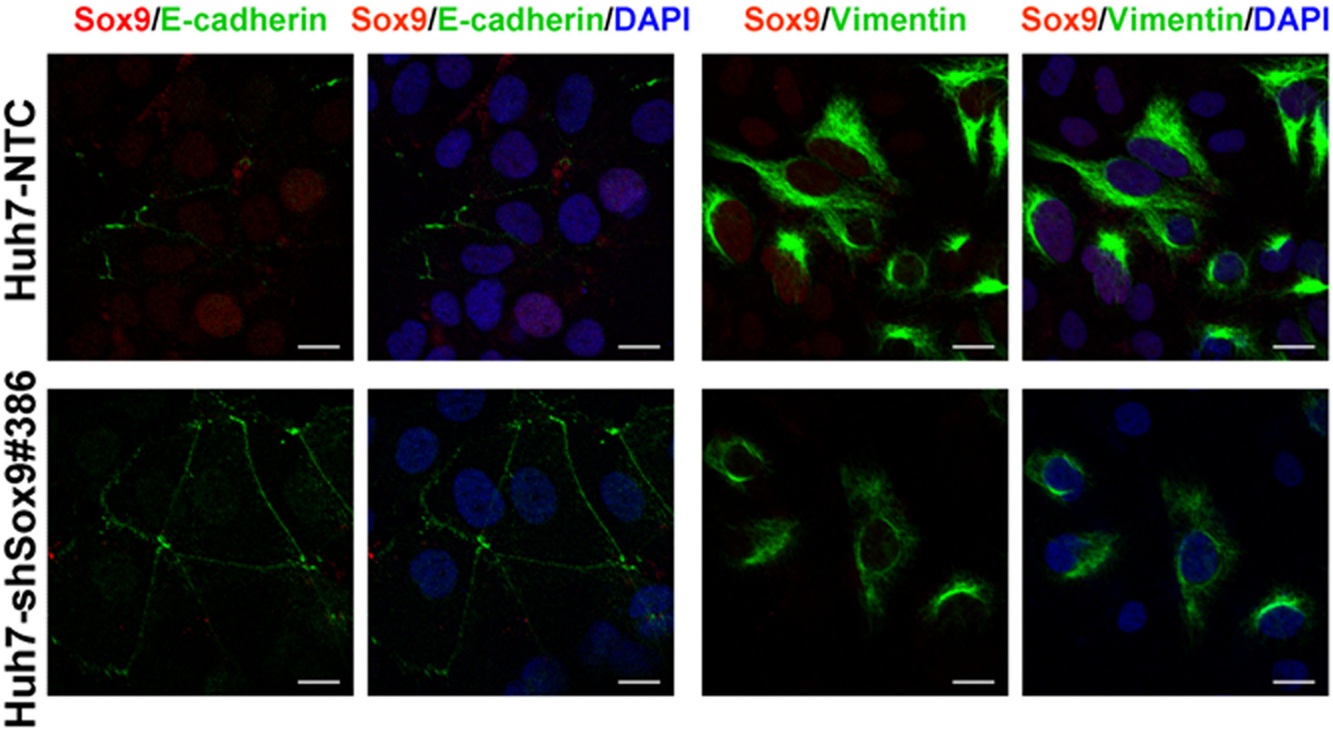

Supplementary Figure S3: Silencing of Sox9 suppressed epithelial-mesenchymal transition. By immunofluorescence, knockdown of Sox9 upregulated E-cadherin expression and downregulated vimentin expression (Scale bar: 50 μm).

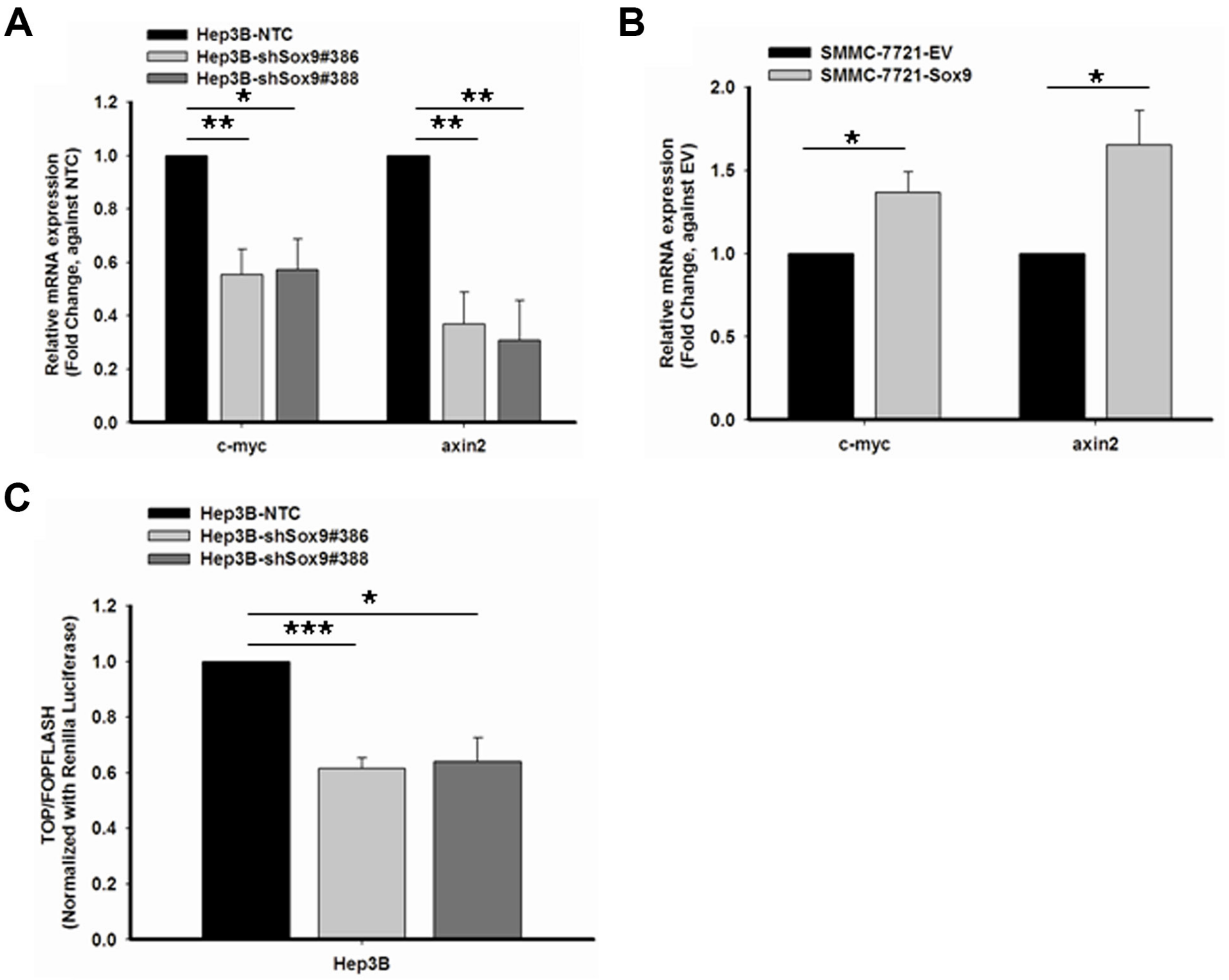

D

No. of cells	Latency (days)		
	NTC+EV	shSox9#386+EV	shSox9#386+β-catenin
$5X10^3$	51.4± 2.2	67.9 ±2.6	61.3 ±2.7
$1X10^4$	47.6±4.7	56.8±2.9	49.0±5.0

Supplementary Figure S4: Sox9 confers stemness features of HCC through Wnt/β-catenin signaling. (**A**) Sox9 silencing in Hep3B suppressed the mRNA levels of c-myc and axin2 ($n = 3$, $*p < 0.05$ & $**p < 0.01$, *t* test). (**B**) Opposite results were observed upon Sox9 forced expression in SMMC-7721 ($n = 3$, $*p < 0.05$, *t* test). (**C**) Knockdown of Sox9 suppressed the activation of Wnt/β-catenin signaling cascade in Hep3B using TOP/FOPFLASH luciferase assay ($n = 3$, $*p < 0.05$ & $***p < 0.001$, *t* test). The data were presented as mean ± SD. (**D**) Enforcement of β-catenin shortened the tumor latency period in Sox9 suppressed group by subcutaneous inoculation model in NOD/SCID mice (results from two independent experiments for 5×10^3 group).

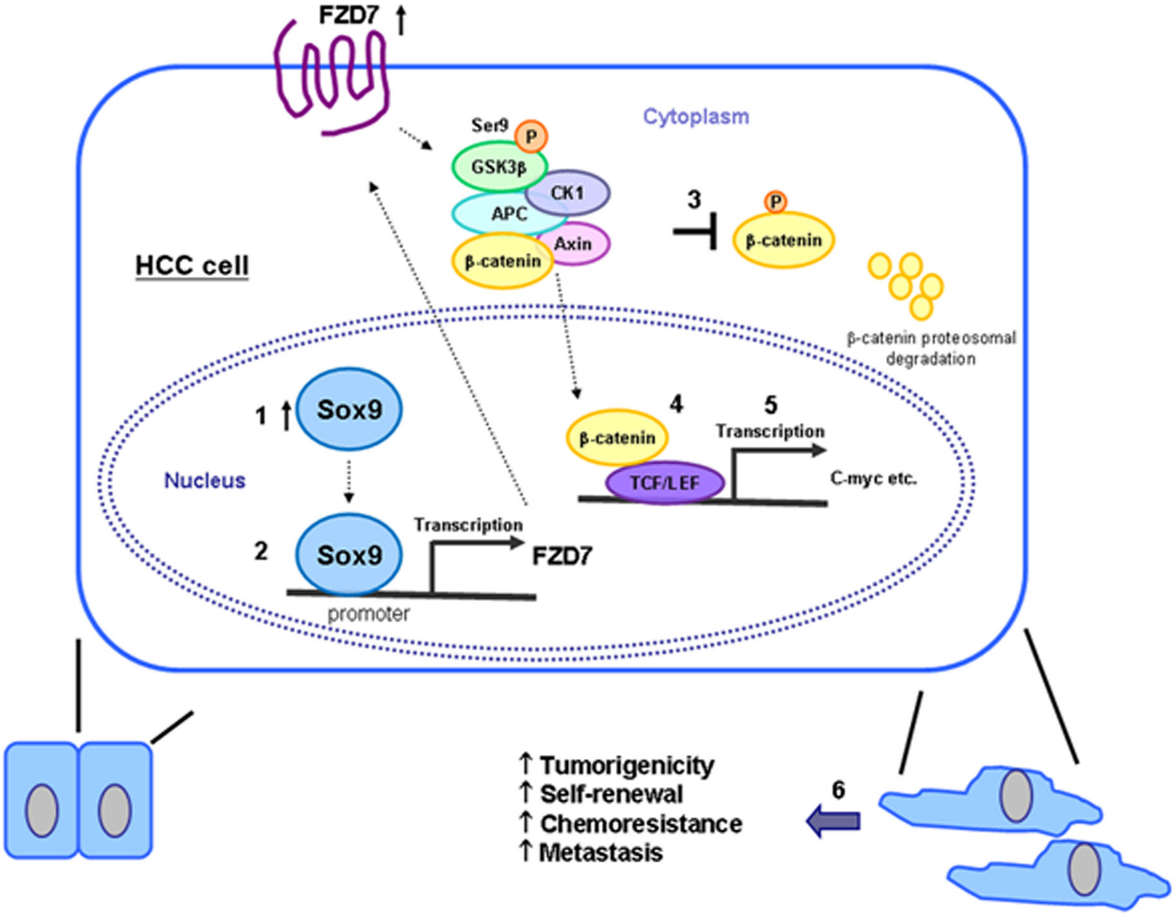

Supplementary Figure S5: Molecular mechanism of deregulated Sox9 in HCC. (**1**) Upregulation of Sox9 in HCC enhances (**2**) the transcription and activity of FZD7 receptor, which in turn (**3**) inhibits β-catenin degradation, possibly through inhibition of GSK3β activity via phosphorylation at serine 9 position. (**4**) The accumulated β-catenin in cytosol is translocated into the nucleus and binds to TCF/LEF, (**5**) activating the transcription of the downstream targets of Wnt/β-catenin signaling pathway. (**6**) The molecular deregulation modulates tumorigenicity, self-renewal ability, chemoresistance capacity and metastasis in HCC.
